# Impact of media components from different suppliers on enterokinase productivity in *Pichia pastoris*

**DOI:** 10.1186/s12896-021-00681-y

**Published:** 2021-03-07

**Authors:** Ján Krahulec, Martin Šafránek

**Affiliations:** Department of Molecular Biology, Comenius University in Bratislava, Faculty of Natural Sciences, Mlynská dolina, Ilkovičova 6, 842 15 Bratislava 4, Slovak Republic

**Keywords:** *Pichia pastoris*, Enterokinase, Enzyme activity, Optimisation, Media

## Abstract

**Background:**

The aim of this study was to provide an information about the homogeneity on the level of enterokinase productivity in *P. pastoris* depending on different suppliers of the media components.

**Results:**

In previous studies, we performed the optimisation process for the production of enterokinase by improving the fermentation process. Enterokinase is the ideal enzyme for removing fusion partners from target recombinant proteins. In this study, we focused our optimization efforts on the sources of cultivation media components. YPD media components were chosen as variables for these experiments. Several suppliers for particular components were combined and the optimisation procedure was performed in 24-well plates. Peptone had the highest impact on enterokinase production, where the difference between the best and worst results was threefold. The least effect on the production level was recorded for yeast extract with a 1.5 fold difference. The worst combination of media components had a activity of only 0.15 U/ml and the best combination had the activity of 0.88 U/ml, i.e., a 5.87 fold difference. A substantially higher impact on the production level of enterokinase was observed during fermentation in two selected media combinations, where the difference was almost 21-fold.

**Conclusions:**

Results demonstrated in the present study show that the media components from different suppliers have high impact on enterokinase productivity and also provide the hypothesis that the optimization process should be multidimensional and for achieving best results it is important to perform massive process also in terms of the particular media component supplier .

## Background

Many approaches were associated with the efficient production of recombinant proteins. The composition of the media is one of the major attributes that affects the economics of cultivation processes and may have a significant impact on production cost and quality. It is generally known that complex media are more favourable for the growth of microorganisms, as their components are directly utilizable in the metabolic pathways, thus saving energy for precursor formation [1]. The difference between complex and defined media at the production level is often notable [2–5].

For example, in the case of *E. coli*, Uderwood et al. [6] demonstrated that the growth rates of genetically engineered strains producing pyruvate decarboxylase and alcohol dehydrogenase are substantially reduced in a defined medium compared to a complex medium. In order to achieve the efficient production and purification of recombinant cysteine proteinase, Aoki et al. [7] firstly cultivated *P. pastoris* in a complex medium to quickly obtain the sufficient amount of biomass and then transferred it to the defined medium.

On the other hand, the overexpression of recombinant proteins directly reduces cell growth by the “protein burden” [8]. The burden which was studied on recombinant strains of *S. cerevisiae* expressing *T. reesei* xylanase, was much higher than expected from the expression level. The supplementation of media (for example with amino acids or succinate) can partially solve the problem, [9]. This observation supports the theory about the demand for amino acids of overproducing recombinant yeast strains [10]. A similar observation was obtained by the study of *Pichia stipites* by Görgens et al. [9]. In this case, the addition of some amino acids to the media improved the production level of xylanase under the control of an oxygen regulated promoter.

Zhang et al. [11] demonstrated that the nitrogen source can play an essential role in the production of heterologous proteins and that the composition of the yeast extract, as a nitrogen source, significantly affects recombinant protein production. They also showed that the composition of yeast extracts varied from lot to lot and the differences resulted in different recombinant protein expression levels [11]. Furthermore, the carbon source also seems to play an important role in terms of productivity and biomass accumulation [12].

The aim of this work was to determine the impact of individual components of YPD media in terms of the component supplier for the production of a heterologous well-characterized and relevant enzyme, the light chain of human enterokinase (EC 3.4.21.9) (hEK_L_) in *P. pastoris* on the laboratory scale. Such knowledge would ensure the avoidance of low enzymatic activity of the expressed protein and achievement of the exact nutrient availability during the cultivation process. As a result, this optimization process would guarantee high cell density and adequate expression levels of the studied protein for further application, for example, in therapeutics and industry.

## Results

**Fig. 1:**
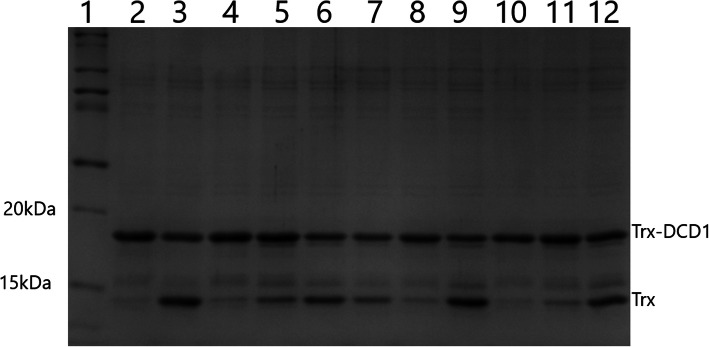
Different culture media and different emzyme activities. Lane 1: protein molecular weight marker. Lane 2: substrate for enterokinase activity Trx-DCD1 as a control. Lane 3: substrate Trx-DCD1 cleaved with EKMax™. Lanes 4–12: Trx-DCD1 cleaved with hEK_L_ expressed in different media composition (media 1 to 9 according Table 2)

The results of this study were obtained from the expression of recombinant hEK_L_ in the *Pichia pastoris* expression system in YPD cultivation medium consisting of different YEs, peptones and glucose suppliers as described in the section Methods. Each particular experiment was carried out at least 4 times. After the expression period, the specific hEK_L_ activity was measured in all media combinations. The results showed a significantly variable cleavage profile (Fig. 1), which indicates that the composition of media ingredients differs depending on the supplier. Trx-DCD1 with a linker recognition sequence for enterokinase (DDDDK↓X) was used as a substrate. We applied the EKMax™ enterokinase as a standard for the experiments as stated in Methods section, and based on the number of experiments, it was determined that 55% substrate digestion (Trx-DCD1) represents 1 unit of the commercial enzyme. From the cleavage percentage of the individual samples, it was possible to calculate the number of enterokinase units in each medium per defined volume, in other words, the enzyme activity. The results from this experiment were used for comparative studies.

**Fig. 2:**
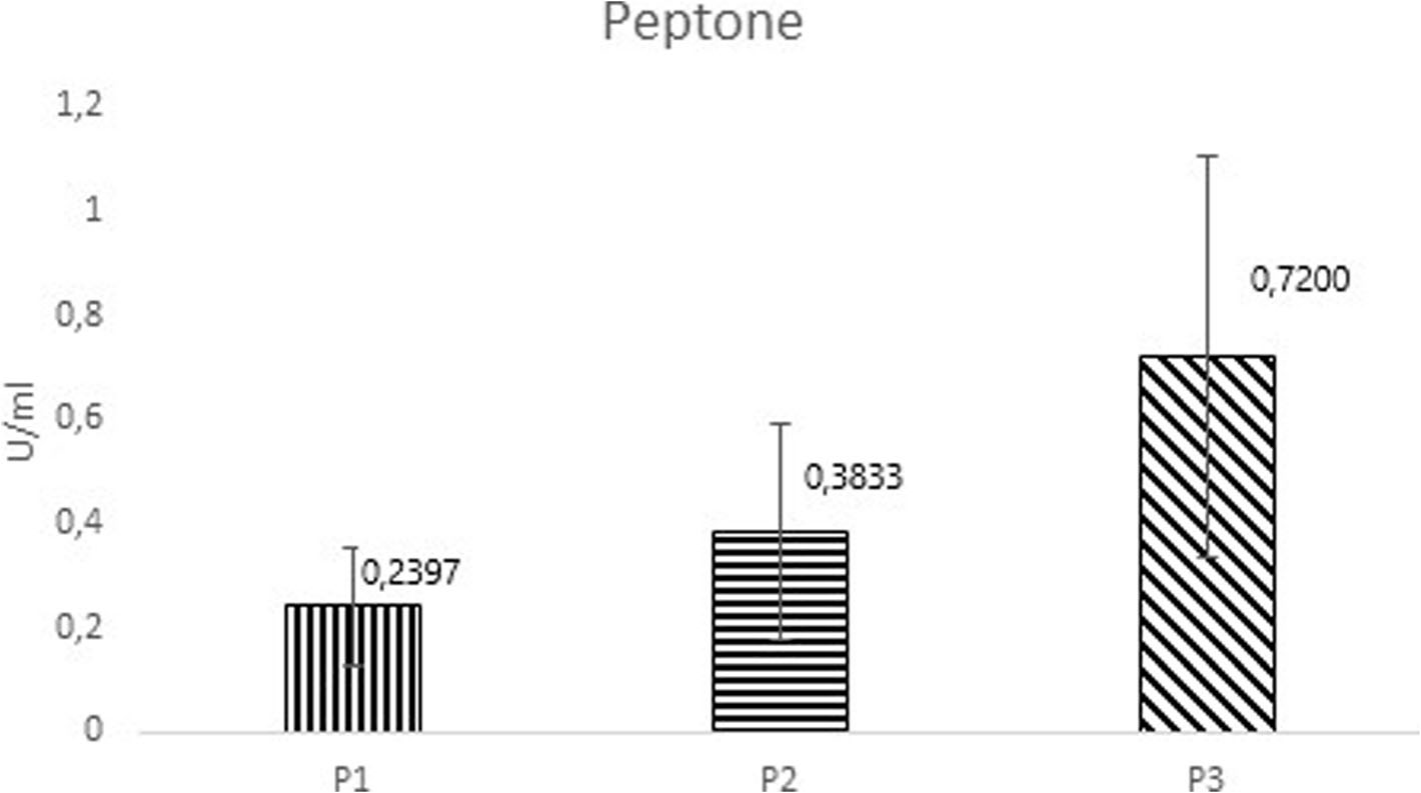
Dependence of the enzyme activity on type of peptone in the medium. P1 represents Sigma-ALDRICH, P2 represents SERVA and P3 represents HIMEDIA

The comparison of the peptones is shown in the Fig. 2. Each peptone used in the common media formulation had apparent impact on the level of expressed hEK_L_. The graph shows the level of enzyme activity relative to the peptone. The enzyme activity of hEK_L_ expressed in media with peptone from Sigma-ALDRICH reached relatively a very low activity level compared to media with peptone from HIMEDIA. The result showed that enterokinase activities in media with peptone from HIMEDIA, SERVA and Sigma-ALDRICH were 0.7200, 0.3833 and 0.2397 U/ml, respectively. There was as much as a 3-fold difference between the average of the highest and the lowest measured enzyme activity. In summary, the media with HIMEDIA peptone showed a 3-fold higher enzyme activity of hEK_L_ than the media with Sigma-ALDRICH peptone.

**Fig. 3:**
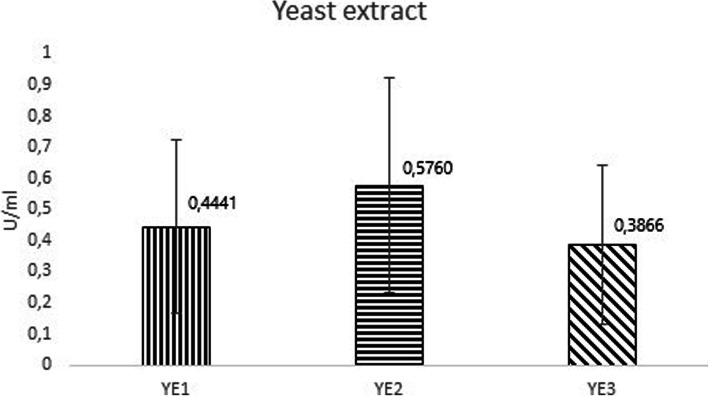
Dependence of the enzyme activity on type of yeast extract in the medium. YE1 represents Sigma-ALDRICH, YE2 represents IMUNA and YE3 represents BIOLIFE

The differences in chemical composition in YE had also apparent effects, similarly as it was for peptone, on the enzyme activity in media. After evaluating the cleavage profile, the results showed that hEK_L_ activities in cultivation media composed of yeast extract from different suppliers (Fig. 3) were 0.5760, 0.4441 and 0.3866 U/ ml for IMUNA, Sigma-ALDRICH, and BIOLIFE, respectively. The individual combinations varied, but ultimately yeast extract alone had the least effect on enterokinase activity in media. The comparison of averages of the lowest and the highest enzyme activities represented a 1.49-fold difference. The results indicate that the IMUNA YE is the most beneficial for enterokinase production among these three YEs.

**Fig. 4:**
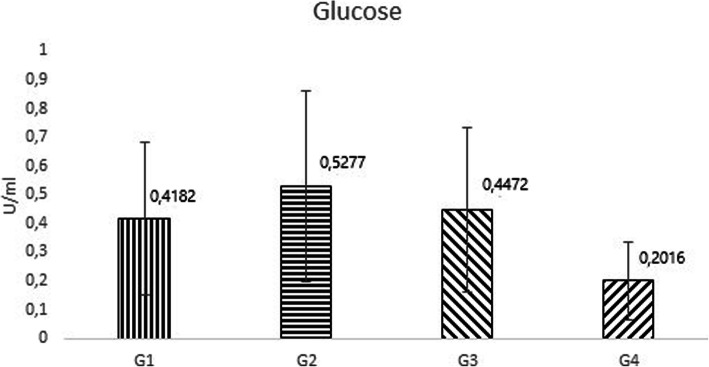
Dependence of the enzyme activity on type of glucose in the medium. G1 represents CENTRALCHEM nonhydrate, G2 represents SERVA, G3 represents CENTRALCHEM hydrate and G4 represents Applichem

Figure 4 shows the level of the enzyme activity of the EK with respect to the used glucose in the media. The enzyme activity in media with glucose from SERVA supplier was 0.5277 U/ ml, CENTRALCHEM (D-glucose anhydrous) 0.4472 U/ ml, CENTRALCHEM (D- glucose hydrate) 0.4182 U/ ml and Applichem 0.2016 U/ ml, on average. From these results, we concluded that glucose from Applichem had the most significant effect on enzyme activity, with activities in some cases ranging up to 4 times lower. Other media composed of glucose from other suppliers had a relatively comparable profile. The difference between the lowest and the highest average activities, in the case of glucose, was 2.62-fold.

**Fig. 5:**
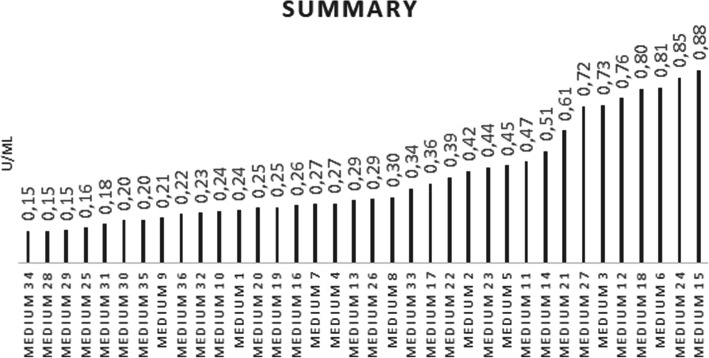
Dependence of enzyme activity on the type of media

The last graph shows the individual media sorted from the lowest to the highest activity levels (Fig. 5). The results varied significantly not only depending on one component, but on the combination of all three components of the YPD cultivation medium. The final analysis showed a 5.87-fold difference between the best and the most adverse combinations in terms of EK activity. After the comparative studies were conducted, it was possible to determine that the worst combination consisted of YE from BIOLIFE, peptone from SERVA and glucose from Applichem, and the most beneficial combination consisted of YE from IMUNA, peptone from HIMEDIA and glucose from SERVA.

In our next step, we proceeded to cultivation experiments in a small bioreactor. We selected two out of a total of thirty-six media composition combinations far enough apart in terms of enzyme activity in the media. After preparing the cultivation medium as described in the Methods, we set the appropriate parameters and initiated cultivation. We tried to maintain constant conditions throughout the entire process for both selected combinations and all replicates. Briefly, in the first phase of fed-batch cultivation, we tried to keep the specific growth rate close to its maximum by regulating the feeding of the feed medium manually, until we reached the maximum technical capabilities of the device. It means stirring 2000 rpm and aeration 4 vvm (volume to volume per minute) with a maximum feeding rate of 0.36–0.42 ml/ min. When the cultivation reached these conditions, we kept DOT (dissolved oxygen tension) nearly below 60%, and decreasing the feeding rate to 0.15 ml/ min. After 144 h of cultivation, the medium with produced enterokinase was separated from the biomass and analysed for cleavage profile.

**Fig. 6:**
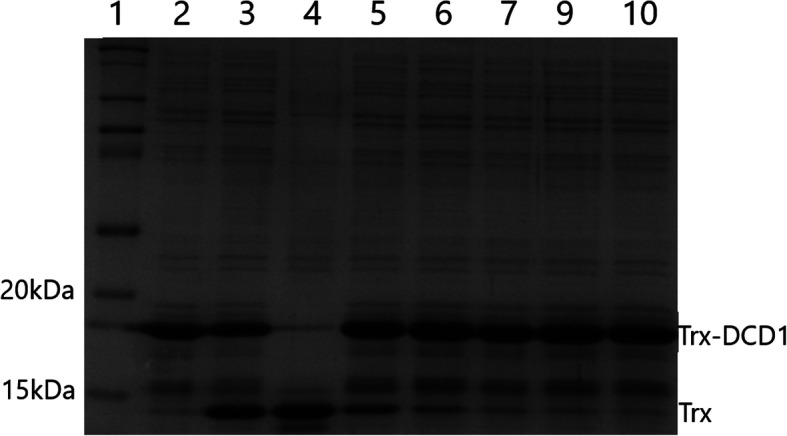
SDS-PAGE analysis of the representative cleavage profile of the medium P1, Y1, G3 after fermentation. Lane 1: protein marker. Lane 2: substrate for enterokinase activity Trx-DCD1 as a control. Lane 3: Trx-DCD1 cleaved with EKMax™. Lanes 4–9: Trx-DCD1 cleaved with undiluted medium, 100x diluted, 300x diluted, 900x diluted, 2700x diluted and 8100x diluted, respectively

**Fig. 7:**
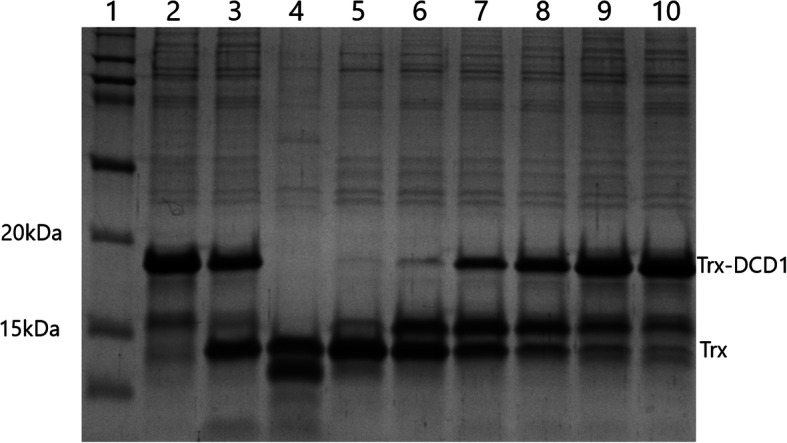
SDS-PAGE of the representative cleavage profile of the medium P3 Y3 G2 after fermentation. Lane 1: protein marker. Lane 2: substrate for enterokinase activity Trx-DCD1 as a control, Lane 3: substrate Trx-DCD1 cleaved with EKMax™, Lanes 4–10: Trx-DCD1 cleaved with undiluted medium, 100x diluted, 300x diluted, 900x diluted, 2700x diluted, 8100x, 24,300x diluted, respectively

**Fig. 8:**
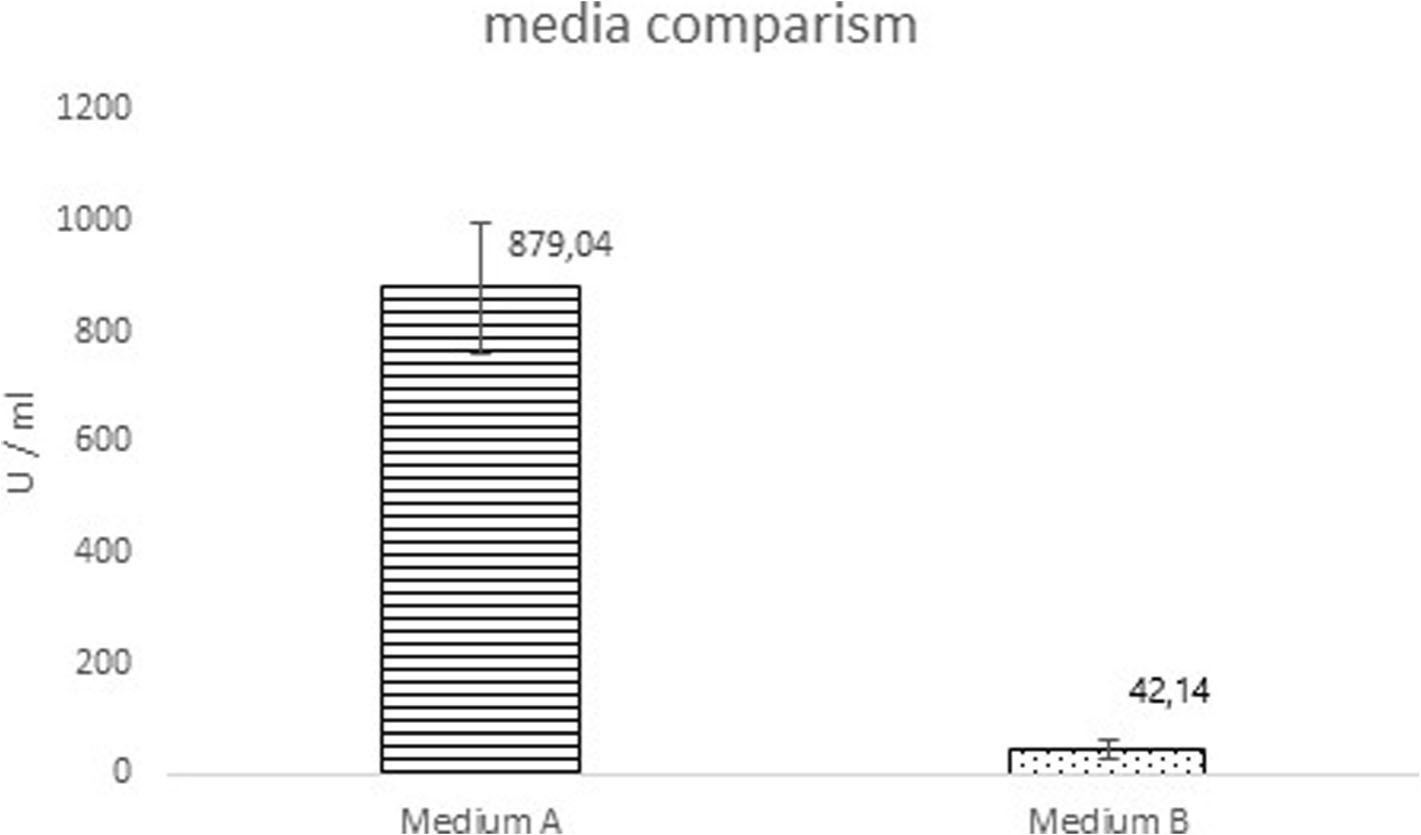
The comparison of enterokinase activity in two media selected for the fermentation experiment

For this experiment we chose media 19 and 18, not the best and worst ones, because YE from IMUNA was not available on the market yet and two relevant glucoses were disposable for our purposes in sufficient amounts for the fermentation at that moment. The difference between them in small volume experiments (2 ml) was 320%. We calculated the enzyme activity for worse combination (P1, Y1, G3) from the cleavage profile on SDS-PAGE (Fig. 6), and it was 42.14 U/ml and 15,588 U in total per fermentation. In the case of the better medium (P3, Y3, G2), the activity was many times higher, which is also evident from the result of SDS-PAGE electrophoresis (Fig. 7). The conversion, in this case, amounted to 879.04 U/ml, with a total volume of 371,544 U (Fig. 8). Ultimately, it represents almost 21-fold difference.

## Discussion

The main aim of this work wasn’t to determine the impact of the media supplements as such, but to determine the sources, or better said, suppliers of the individual media components and thus provide perspective on finding the right supplement suppliers for the effective production of a recombinant light chain of human enteropeptidase in yeast *Pichia pastoris* production system, which would prove higher yields and higher activities. Furthermore, this study strives to draw attention to the fact that it is worth performing the optimisation process in terms of finding the proper suppliers of the media components.

A peptone is composed of short polypeptides, peptides and amino acids that typically originate from a broken commodity protein by an enzymatic digestion. After the process of digestion, the mixture is further purified and processed into a dry powder. Such process ensures that the components of the peptone are then more available as nutrient requirements, like nitrogen and carbon, making the peptone suitable for the formulation of cultivation media for microorganisms [13].

Peptones, in general, are very popular as the components for preparing different media. Several aspects of a peptone can be taken into consideration in terms of promoting growth activities. It can support intensive cell growth at the beginning, providing a sufficient amount of the nutrients on one hand; however, such intensive growth at the beginning leads to a depletion of the vital nutrients and the subsequent production of metabolites, which can inhibit cell growth [14].

The intent of the current study and other papers [15] was to determine the effect of peptone and other elements on the production level of active proteins. The results presented in this paper demonstrated the enzyme activity dependent on the peptone and varied from 0.2396 U/ ml to 0.7200 U/ ml. These activities were substantially different, had a major impact on enzyme activity, and thus, we can safely say that peptones from different suppliers were involved in achieving higher enzyme activity in the medium after cultivation.

Although the positive effect of peptone is not fully understood at the molecular level, it can be concluded in general that it is a consequence of heterogeneous amino acid composition of the peptones [16–18].. However, the relation between their amino acid content and their role in the growth, production, and metabolic behaviour of recombinant cells is poorly understood. Source-dependent differences for the enzyme yields of growth cultures were evident. This observation again indicated that different manufacturing techniques between companies result in a distinct product composition, which were variable in their ability to support microorganism growth or requirements for overproduction of recombinant products. The ability of different peptones to affect the cultivation was demonstrated by Brãnes et al. [19] and these variations were considered to be associated with the digestive process of peptone manufacture.

The selection of a nitrogen source to produce heterologous proteins is crucial and it was demonstrated by observations with various complex nitrogen sources for recombinant protein production by Van Niel et al. [20] and Miller [21]. Heterogeneity in complex components, such as yeast extract, can result in reduced reproducibility of expression performance, as shown by Zhang et al. [11], where the heterologous protein production levels differed by 2–3 fold. In our study, we observed the effect of YE on the activity of the enzyme, which ultimately did not represent large differences with the three used suppliers, and the activity fluctuated from 0.386 to 0.575 U/ml. Rose and Harrison [22] reported that yeast extract could be used as a complex additive to bring trace elements and growth factors into the culture medium at low costs. However, there is still an urgent demand for systematically exploring the molecular mechanisms. The presence of nitrogen components in cultivation media may also be important to protect heterologous proteins in the extracellular medium from proteolysis. Extracellular proteolysis of heterologous proteins is affected by nutritional conditions and may increase due to glucose exhaustion or carbon starvation [23, 24].

In a comparative study of individual glucose, we achieved relatively similar results except for one glucose from the supplier Applichem, where the value of the enzyme activity in some cases had a 4-fold difference. Several transcriptomic analyses have focused on the response to high sugar stress on protein expression. Kaeberlein et al. [25] described an increase in the expression of glycerol and trehalose biosynthetic genes in cells exposed to 20% (w/v) glucose. In a more recent study [26]), an expression higher than 2-fold was also found under conditions with a glucose additive for genes that participate in response to chemical stimuli. Glucose is the only medium component from the three used in our study that is chemically defined. The supplier defines the content of glucose in a minimum amount, for example 99%, and other components (heavy metals, chloride, sulphate etc.) in a maximum amount, but not in an exact manner. And it is probably this residual content, aside from the glucose, which has a major impact on glucose quality for the cultivation processes.

## Conclusions

The fermentation experiment showed that controlled cultivation is much more sensitive to the component source than small volume laboratory experiments. Even for laboratory purposes, the differences shown in this study are enormous, confirming the importance of choosing the right media component suppliers. This study has shown the importance of the optimization process also in terms of the media component supplier. This study does not show the universal combination of the component sources but tries to illustrate that the optimization process is multi-dimensional and every recombinant product in any host probably requires a massive individual optimization process also in terms of the media component supplier. Moreover, it would be interesting to perform a similar study between different batches of the same product of the same supplier, since it is obvious that differences between batches surely exist [11].

## Methods

The *P. pastoris* Y11430 pGAPZαC hEK_L_ strain expressing hEK_L_ under the control of the *P. pastoris* constitutive GAP promoter was used in the experiment [27]. Single colonies of *P. pastoris* were picked up from growth plate and inoculated in glass tubes containing 2 mL of YPD medium (1% (w/v) yeast extract; 2% (w/v) peptone; 2% (w/v) glucose). The pre-growth cultures were incubated at 28 °C with an overnight shaking speed of 200 rpm. Afterwards the cell culture was collected by centrifugation with set parameters of 13,400 rpm and 10 min (Minispin, Eppendorf, rotor F-45-12-11) and the cell pellets were washed three times with sterilized ultrapure water (Milli-Q 0,05^− 1^ μS.cm^− 1^, Millipore). A calculated volume of pre-growth was inoculated on 24-well plates to an OD_600_ = 0.5 starting culture density end volume of 1 ml. Three commercial Yeast extract (YE) powders, three Peptone powders and four glucose sources from different suppliers were used in the cultivation study as shown in table (Table 1) and a total of 36 cultivation combinations were created. For simple orientation purposes, Table 2 provides a list of all combinations used in the experiment. The proprietary glucose source was added to the culture with a final concentration of 2% (w/v) at every 24 h. Cultivations were performed at 28 °C with a shaking speed of 200 rpm for 7 days. After cultivation, the cells were separated from the biomass by centrifugation at the conditions mentioned above.

**Table 1:** Media component sources sorted by suppliers

Mark	Supplier
Y1	92,144 Yeast extract for microbiology Sigma-Aldrich
Y2	Yeast extract 500 g, IMUNA
Y3	4,122,202 Yeast extract BIOLIFE
P1	82,962 Peptone from meat, enzymatic digest, Sigma-Aldrich
P2	48,619.02 Peptone from meat, pancreatic, Serva
P3	RM001 Peptone, bacteriological, HIMEDIA
G1	L-00988 D-Glucose anhydrous, CENTRALCHEM
G2	22,720.02, α-D- Glucose.H_2_O analytical grade, SERVA
G3	L-03329, D-Glukose hydrate, CENTRALCHEM
G4	A1349,1000; D(+)-Glucose 1-hydrate, Applichem

**Table 2:** Media combinations used in the experiment

Mark	Medium composition	Mark	Medium composition	Mark	Medium composition
1	G1-Y1-P1	13	G2-Y2-P1	25	G3-Y3-P1
2	G1-Y1-P2	14	G2-Y2-P2	26	G3-Y3-P2
3	G1-Y1-P3	15	G2-Y2-P3	27	G3-Y3-P3
4	G1-Y2-P1	16	G2-Y3-P1	28	G4-Y1-P1
5	G1-Y2-P2	17	G2-Y3-P2	29	G4-Y1-P2
6	G1-Y2-P3	18	G2-Y3-P3	30	G4-Y1-P3
7	G1-Y3-P1	19	G3-Y1-P1	31	G4-Y2-P1
8	G1-Y3-P2	20	G3-Y1-P2	32	G4-Y2-P2
9	G1-Y3-P3	21	G3-Y1-P3	33	G4-Y2-P3
10	G2-Y1-P1	22	G3-Y2-P1	34	G4-Y3-P1
11	G2-Y1-P2	23	G3-Y2-P2	35	G4-Y3-P2
12	G2-Y1-P3	24	G3-Y2-P3	36	G4-Y3-P3

Fermentation was performed according to Melicherová et al. [28], i.e. fermentations were carried out in a 2 l bioreactor (2 l Sartorius Biostat B+) containing 750 mL of the fermentation medium. Unless the conditions were modified, the medium contained 26.7 ml.l^− 1^ H_3_PO_4_, 5.6 ml.l^− 1^ H_2_SO_4_, 15.9 g.l^− 1^ KOH supplemented with 10 g.l^− 1^ of yeast extract and 20 g.l^− 1^ meat pepton appropriate for the selected media combination (P1, Y1; P3, Y3). The seed culture was performed in a 250 mL Erlenmayer flask with a working volume of 50 mL at 28 °C for 24 h, 180 rpm. The inoculation medium consisted of 13.8 g.l^− 1^ (NH_4_)_2_SO_4_, 46 g.l^− 1^ (NH_4_)H_2_PO_4_, 15.9 g.l^− 1^ KOH, 10 g.l^− 1^ yeast extract and 20 g.l^− 1^ meat pepton, and as mentioned above, according to the selected media combination. After adjusting pH to 6.0 by H_3_PO_4_ and sterilization, 0.43 ml of the necessary trace element solution PMT1 (0.5 g.l^− 1^ CoCl_2_.6H_2_, 65 g.l^− 1^ FeSO_4_.7H_2_O, 3 g.l^− 1^ MnSO_4_.5H_2_O, 5 ml.l^− 1^ H_2_SO_4_ (95–98%), 0.08 g.l^− 1^ KI, 6 g.l^− 1^ CuSO_4_.5H_2_O, 20 g.l^− 1^ ZnCl_2_, 0.02 g.l^− 1^ H_3_BO_3_, 0.2 g.l^− 1^ Na_2_MoO_4_.2H_2_O and 0.2 g biotin) was added along with 0.4 ml.l^− 1^ of 1 M MgSO_4_, 0.02 ml.l^− 1^ of 1 M CaCl_2_ and 10 g.l^− 1^ glucose appropriate for the selected carbon source (G3, G2).

Before inoculation, 3.2 mL of PMT1 were added to a sterile medium and the bioreactor was set to cultivation parameters (mixing 200 rpm, temperature 29 °C, pH 6, dissolved oxygen tension (DOT) 60%). Selected parameters were controlled during 144 h of cultivation. When the carbon source in the medium was nearly exhausted, the feeding medium (500 ml.l^− 1^ of glucose, 100 mM MgSO_4_ and 5 mM CaSO_4,_ after sterilization was added 12 ml.l^− 1^ PMT1 solution) was raised to the appropriate level. The aim was to keep the specific growth rate close to, but not at the maximum. After reaching the maximum technical parameters of the fermenter in rotation and aeration, we kept the manual settings at 60% DOT by lowering the feeding rate. The medium was separated from biomass after cultivation at 7000 rpm at 4 °C (centrifuge Sorval RC 6+).

The enzymatic activity of hEKL secreted into the medium during cultivation processes was measured by the digestion of Trx-DCD1 (thioredoxin-DDDDK-dermcidin 1). The reaction mixture with a total volume of 5 μl consisted of 10 mM Tris-HCl pH 8.0, 4 mg.ml^− 1^ Trx-DCD1 and hEK_L_ or the enzymatic standard. The cleaving reaction was performed for 1 h at 37 °C and was stopped by the addition of 20 μl of SDS-PAGE sample buffer (62.5 mM Tris-HCl pH 6.8, 1% (v/v) glycerol, 2% (w/v) SDS, 0.00125 (w/v) bromphenol blue, 5% (v/v) 2-mercaptoethanol). The cleavage was analyzed by 16% SDS-PAGE. The SDS-PAGE protein separation was visualized by Coomassie Brilliant Blue R250 colloidal staining and the cleavage analysis was performed using densitometric software (Genetools, Syngene, UK). We used commercial bovine enterokinase EKMax™ (ThermoFisher Scientific) with a specific activity of 2000 U.mg^− 1^ as the standard.

## Data Availability

All data generated or analysed during this study are included in this published article.
